# Using the Constrained Disorder Principle to Navigate Uncertainties in Biology and Medicine: Refining Fuzzy Algorithms

**DOI:** 10.3390/biology13100830

**Published:** 2024-10-16

**Authors:** Yaron Ilan

**Affiliations:** Department of Medicine, Hadassah Medical Center, Faculty of Medicine, Hebrew University, Jerusalem 9112001, Israel; ilan@hadassah.org.il

**Keywords:** uncertainties, digital systems, fuzzy algorithms, biological noise, constrained disorder principle, biological variation

## Abstract

Uncertainty in biology refers to situations in which information is imperfect or unknown. Variability, on the other hand, is measured by the frequency distribution of observed data. Biological variability adds to the uncertainty. The Constrained Disorder Principle (CDP) defines all systems in the universe by their inherent variability. According to the CDP, systems exhibit a degree of variability necessary for their proper function, allowing them to adapt to changes in their environments. Per the CDP, while variability differs from uncertainty, it can be viewed as a regulated mechanism for efficient functionality rather than uncertainty. This paper explores the various aspects of un-certainties in biology. It focuses on using CDP-based platforms for refining fuzzy algorithms to address some of the challenges associated with biological and medical uncertainties. Developing a fuzzy decision tree that considers the natural variability of systems can help minimize uncertainty. This method can reveal previously unidentified classes, reduce the number of unknowns, improve the accuracy of modeling results, and generate algorithm outputs that are more biologically and clinically relevant.

## Simple Summary

Uncertainty in biology refers to situations in which information is imperfect or unknown. Variability is measured by the frequency distribution of observed data, allowing for an understanding of the fundamental principles influencing diversity across different levels of biological organization. Biological variability adds to the uncertainty. The Constrained Disorder Principle (CDP) defines all systems in the universe by their inherent variability. Per the CDP, while variability differs from uncertainty, it can be viewed as a regulated mechanism for efficient functionality rather than uncertainty. This paper examines the different aspects of uncertainty in biology. It specifically looks at using CDP-based platforms to improve fuzzy algorithms and tackle some of the challenges related to biological and medical uncertainties. The goal is to produce algorithm outputs more relevant to biology and clinical applications.

## 1. Introduction

The field of biology is intrinsically uncertain, from the molecular intricacies of cellular processes to the global dynamics of ecosystems. Although biological research has made tremendous progress, uncertainties persist, presenting numerous challenges [1]. Managing unknowns in biology and medicine requires a multifaceted and dynamic approach [2]. The Constrained Disorder Principle (CDP) defines all systems in the universe by their inherent variability.

This paper aims to explore the multifaceted nature of uncertainties in biology and examine their origins, implications, and strategies for effective management. The paper focuses on using CDP-based platforms and refining fuzzy algorithms to overcome the challenges associated with biological and medical uncertainties.

## 2. The CDP Defines Systems in the Universe by Their Degree of Variability

The Constrained Disorder Principle (CDP) defines all systems in the universe by their inherent variability. Per the CDP, living and non-living systems are characterized by a degree of variability, which is mandatory for their proper function and enables them to adapt to the dynamic nature of their environments [3]. The dynamic boundaries of variability determine the degree of variability in a system. These boundaries continuously change the inherent variability, enabling adequate function under fluctuating conditions [3].

The CDP defines system malfunctions as the inadequate functionality of the variability boundaries, leading to excess or insufficient system variability. While regulated by the boundaries, the degree of variability cannot be predicted ahead of time, as it depends on the continuously changing internal and external environments. Per the CDP, this is an active process that works in dynamic environments, ensuring system efficiency. Variability differs from uncertainty. Nevertheless, system variability can be schematically viewed as a built-in uncertainty, representing a sophisticated regulated mechanism for efficient functionality.

The CDP applies to all systems and is reflected in human behavior and decision making. It is schematically described by the B = F formula, where B stands for borders and F for functionality and efficiency. The formula illustrates the concept that dynamic borders regulate the degree of variability in a system, thus determining its performance [4].

## 3. The CDP Accounts for Quantum-Based Variability

Determinism means that the fundamental laws of physics govern the universe’s state of history and evolution [5]. The concept of ‘strong determinism’ implies that the past determines the future and that the universe’s history is permanently fixed. Only one track is laid down in strong determinism, no matter where it starts [5]. Implementing strong determinism in classical physics is challenging. Despite the simplicity of classical physics’ dynamical laws, the universe is complex, and its initial conditions must have also been. The precise positions and momenta of all the particles involved require much information. Any statement of the initial condition is too complex to be a law [6,7]. Classical mechanics is deterministic, because it provides conditional statements about the evolutionary histories of the universe. However, standard determinism does not fully satisfy the principle of sufficient reason, as something remains unaccounted for in the initial state [6]. A comprehensive theory of the universe must be able to predict the large-scale structures of the universe. Newtonian or Einsteinian dynamic equations alone do not accomplish this. The observed phenomena depend significantly on the initial conditions [6,8].

According to Newton’s laws, the past does not determine how objects move in the future. Since there is no upper limit on how much an object can accelerate, a classical object can theoretically reach spatial infinity within a finite time. The reversal of this process implies that objects may originate from spatial infinity without a causal connection to anything else in the universe and cannot be predicted based on the universe’s past states [9]. The speed of light solves this problem. According to the equations of general relativity, there are singularities with infinite curvature, such as black holes in the universe. In certain instances, singularities behave like gaps in space-time where the existing theory no longer applies, allowing anything to emerge from or disappear into them, which poses a threat to determinism [6,10].

Heisenberg’s uncertainty principle describes an imprecision in nature, resulting in a limit to what can be known about the behavior of quantum particles [11]. The principle states that it is impossible to accurately determine a moving particle’s position and momentum simultaneously. This is due to inherent errors in the measurements, and these errors cannot be less than the quantum constant. Although these errors are insignificant on a human scale, they are essential and cannot be disregarded. Essentially, the more precisely we know the position of a particle, the less precisely we can know its momentum, and vice versa [11].

Quantum theory explains the universe’s characteristics better, allowing for only the determination of probabilities [12]. When a wave function is measured, it randomly jumps between two states, and quantum mechanics specifies the probability of each state occurring [6,13]. As a quantum object’s wave function spans many “classical” states, one could propose a simple initial condition that includes all the complexities as emergent structures in the quantum superposition of these states [14]. Observed complexity partially describes the fundamental reality represented by the universe’s wave function [6,15,16]. The quantum universe consists of a simple law that chooses an initial wave function for the universe and is governed by solid determinism [12]. According to the physical laws, there can only be one cosmic history of the universe, described by a wave function combining many classical trajectories. Each event, including the first, is explained as the laws that determine the entire wave function of the universe [17]. Quantum mechanics’ probabilities may not manifest at the level of physical laws but can still be attributed to generalized and partial descriptions of universe components. As ‘superpositions of superpositions’, density matrices provide extra avenues for establishing the universe’s initial state [6,18].

The CDP accounts for quantum variability. This principle applies to microscopic and macroscopic systems and views the variability at the quantum level as part of the inherent variability mandatory for proper function [19]. The CDP provides a unified law describing the unknown uncertainties, variabilities, and noises required for appropriate subatomic particles and macroscopic system functions. Per this principle, the boundaries of the variability are dynamic, enabling systems to adapt to the perturbations and uncertainties around them continuously.

## 4. Uncertainties, Unknowns, Risks, and Variabilities Characterize Complex Systems

Uncertainty refers to situations in which information is imperfect or unknown [20]. It occurs when there is no way to precisely describe the present state, a future outcome, or more than one possible outcome. It applies to predicting future events, existing measurements, or unknowns. Partial observation, stochastic environments, ignorance, or all three, may cause uncertainty.

The impact of an uncertain event on a decision is determined by its probability in normative decision theory. Probabilities are known in some cases and unknown in others. Unknowable uncertainty is an uncertain situation where no one can access the missing information. The absence of information influences the appeal of a bet based on an uncertain event, especially if the information is available to someone else. Unknown uncertainty falls in between known uncertainty and unknowable uncertainty. Unknown uncertainty occurs when the subject does not know the probability but believes someone else may know it. According to unknowable uncertainty, probabilities are unknown to everyone to some reasonable extent [21]. A lack of understanding can be categorized into known unknowns, which are things subjects realize they do not know; unknown unknowns, which subjects do not realize they do not know; and wrong assumptions, which subjects think they know but do not know. Knowing there are unknowns and their potential for generating surprises and black swans indicates that top-down approaches, which predict hazards quantitatively, contrast with bottom-up approaches considering societal vulnerabilities and failure possibilities [20,21]. Knowing there are unknowns and their potential for generating surprises and black swans indicates that top-down approaches, which predict hazards quantitatively, contrast with bottom-up approaches considering societal vulnerabilities and failure possibilities [22,23].

A probability distribution is used based on knowledge about the likelihood of what a single, actual value of an uncertain quantity is for quantifying uncertainty [24]. Possible states or outcomes can be assigned probabilities, which includes applying a probability density function to continuous variables. The probability density function is used in statistics to represent second-order uncertainty over first-order probabilities. The posterior probability distribution can be used to quantify epistemological uncertainty [25].

A risk is a state of uncertainty in which some possible outcomes might result in an undesired outcome or a significant loss [26]. A risk analysis includes a set of measured uncertainties, with some possible outcomes resulting in losses and the magnitude of those losses. Loss functions over continuous variables are also included in Section 11.

The concept of variability is distinct from that of uncertainty. Variability is quantified by the frequency distribution of multiple instances of a quantity derived from observational data [27]. The CDP illustrates systems’ natural variability as an inherent part of their built-in uncertainty, which characterizes complex systems.

## 5. The CDP Distinguishes between Biological Uncertainties, Noise, Variability, and Variation

Differentiating between biological variation, inherent biological noise, and variability is crucial for understanding biological uncertainties and developing methods to deal with them [28]. While variability differs from uncertainty, the CDP considers it a form of inherent uncertainty, representing a controlled mechanism for efficient functionality.

Per the CDP, biological noise and variability must be distinguished from variation, as they affect the accuracy of algorithm outputs and, therefore, should not be disregarded [29]. Different results can be expected when multiple samples from the same individual are measured over time. The CDP states that this variation includes both technical and inherent biological variability. CDP-based modeling eliminates the technical variation that results from measurement techniques and focuses on biological systems’ inherent variability and noise [28]. Biological systems are unpredictable due to the interactions among genes, proteins, and environmental factors. All biological processes are disordered to some degree [29,30,31,32,33,34,35,36,37,38,39,40,41]. It takes a nuanced understanding of biology to deal with uncertainties in the microcosmic realm of molecular biology and the macrocosmic realm of ecology and evolution [42]. The uncertainty of biological systems can be categorized into two types, namely aleatoric and epistemic. Aleatoric uncertainty arises from inherent internal noise in a biological system and can be addressed using stochastic methods like stochastic differential equations, Markov chains, or stochastic Petri nets (SPNs). Epistemic uncertainty results from a lack of knowledge about the system being studied, often due to a limited understanding of the underlying mechanisms, incomplete measurement data for some components, or measurement errors for specific data [43,44]. These two types of uncertainty complicate the modeling and analysis of biological systems. The modeling of biological systems is accompanied by epistemic uncertainties, ranging from structural to parametric uncertainty, due to limitations like an insufficient understanding of the underlying mechanism and incomplete system measurement data [45]. When there is a lack of a complete understanding of how a system works or when sufficient data cannot be obtained, this is known as structural uncertainty. This means it is difficult to determine the exact structure of the model that needs to be constructed. Even when the model’s structure is determined, there is a need to contend with parametric uncertainty due to incomplete or inaccurate measurement data.

Biological noise is the substantial cell-to-cell variability observed in populations of genetically identical cells. It is increasingly recognized as an essential factor in biology [46]. As defined by the CDP, biological variability is part of the universe’s stochasticity and includes biological noise and variability at the genetic, protein, and organ levels, including the brain [4,29,30,31,32,33,34,35,36,37,38,39,40,41,47,48,49,50,51,52,53,54,55]. Genetic variability characterizes the genome of all living systems, and diverse genetic backgrounds lead to varied responses to stimuli, diseases, and environmental changes [48]. Genetic factors influence phenotypic outcomes in personalized medicine. Variability is also inherent in the function of cells and whole organs such as the brain, heart, and immune system [47,56]. The concepts of biological noise and biological system variabilities align with the CDP, which describes systems regarding the dynamic boundaries of their variability [19].

Biological variation refers to the variation in testing, such as serum levels of biochemical analytes. It can occur within a single subject or between subjects [57]. Per the above definition of noise and variability, biological variation comprises the technical variation between labs and measuring devices and the tested system’s inherent variability. Biological variation data are obtained through various study designs, including experimental studies and analyses of routine laboratory results from laboratory databases [58]. It is possible to apply biological variation data to laboratory applications, including setting analytical performance specifications, determining the optimal sample for analyzing a specific constituent, assessing population-based reference ranges’ usefulness, and assessing variation in serial results from an individual [59]. Applications of biological variation data include setting analytical performance specifications, calculating reference change values, defining the index of individuality, and establishing personalized reference intervals. Biological variation data are derived using new models, including the Biological Variation Data Critical Appraisal Checklist (BIVAC), the highly powered European Biological Variation Study (EuBIVAS), the EFLM Biological Variation Database, and new applications of the data, such as personalized reference intervals and measurement uncertainty [58].

An individual’s variation arises from the imprecision of the measurement procedure and the rhythmic and random fluctuations of quantity values around a virtual homeostatic set point, the intra-individual biological variation. Based on the intra-individual variation in a quantity, a mean value, the virtual homeostatic set point, is calculated for each individual. The biological variation among individuals causes the variation between these mean values [60]. Each individual has a “subject mean”, a “central tendency”, a “control level”, or a “set point” concentration for maintaining homeostasis, which is influenced by genes, diet, exercise, and age [60]. Biological variation information helps establish reference intervals based on population, assess the significance of serial findings, and analyze correctness [47]. Within- and between-subject biological variation, the index of individuality, and the reference change value is essential to determine biochemical analytes. These parameters were calculated for many serum biochemistry analytes. These analytes, blood sugar, creatinine, urea, uric acid, sodium, potassium, chloride, magnesium, and phosphate, show low individuality. However, calcium has high individuality, suggesting that population-based reference intervals are more appropriate for determining it [61].

Microarray and RNA sequencing data show that expression measurements are biologically variable across individuals, regardless of the measurement technology used. Analyses designed to estimate this variability produced more reproducible RNA sequencing results [62]. For biological measurements, the reference point is a clinical decision point based on a trial or a patient’s past results. Quality control and external quality assurance limits were set using biological variation. Patient-based real-time quality control and patient-based quality assurance are currently used to detect changes in variation assay performance [63].

These examples highlight the challenges of quantifying and differentiating biological variabilities from biological variation. While current techniques for using variation data help reduce “measurement technique-related variation”, it is still challenging to quantify the inherent biological variability [28].

Heisenberg’s uncertainty principle states that measuring a particle’s variables involves inherent uncertainty. Commonly applied to the position and momentum of a particle, the principle states that the more precisely the position is known, the more uncertain the momentum, and vice versa [11]. Heisenberg’s uncertainty principle has been proposed to apply to numerous areas. The genetic information that drives the function of living cells is best represented by a probabilistic model rather than as a wholly defined object. A formal description of a cell’s genetic information should include the number of DNA molecules in that cell and their complete nucleotide sequences. It is impossible to know the genome sequence of any living cell with absolute certainty, as there is always some associated uncertainty, especially regarding base pairs and other functions [64].

Per the CDP, biological variability, like the variability of all systems, is essential for proper function [49,50,51,52,53,54,65]. The CDP states that personalized variability demonstrates phenotypic variability indicators, encompassing genetic and all other forms of variability. Personalized variability indicators illustrate the impact of ongoing disturbances on the dynamic boundaries of variability and, therefore, consistently differ within and between subjects [66].

## 6. The CDP and Evolutionary Dynamics

Evolutionary processes depend on dissimilarities, enabling organisms to persist and adapt to changing environments [67]. Biological systems differ ubiquitously. Various factors shape evolutionary processes, including selective pressures, genetic mutations, and environmental shifts. The interactions between ecological and genetic factors can predict the trajectory of evolution and its impact on species adaptations [68]. Ecosystems constantly change, impacting biodiversity and environmental balance, which pose significant challenges [69].

Heisenberg’s uncertainty principle in quantum mechanics is fundamental to developing evolutionary variability [70]. When this principle is considered alongside the conservation of energy and material resources principle, it leads to a relationship of uncertainty between local fluctuations in the quantities to be conserved on a global scale and the rate of their regional variation. The uncertainty relationship is reflected in local fluctuations associated with a non-zero rate of variation, which gives rise to subsequent volatility. The generative nature of the uncertainty relationship is not random. It is prevalent through various evolutionary stages, from the abiotic synthesis of monomers and polymers to the emergence of behavior-induced variability in organisms [70].

The CDP does not consider processes in the universe as temporal. Living and non-living systems are machine-like platforms with built-in mechanisms that enable them to adapt to noisy environments. They are not intentionally designed and do not desire improvement. Atoms do not have aspirations. The boundaries of variability contain a “memory” of past events, representing the range of adaptability in the system due to repeated responses to disturbances [19].

## 7. The CDP Defines Decision-Making under Uncertainties

An uncertain event’s impact on a decision is determined by its probability in normative decision theory. It is impossible to accurately capture people’s reactions to uncertainty with a single notion of probability [71]. Probabilities are known in some cases and unknown in others. An unknowable uncertainty is an uncertain situation where no one can access the missing information. The absence of information influences the appeal of a bet based on an uncertain event, especially if the information is available to someone else [21,72]. A relative disregard for uncertainty arises from possibilities beyond imagination, which cannot be considered probabilistic [73].

People should actively seek out uncertain options in repeated decision problems where they can learn from experience rather than avoid ambiguity and uncertainty to improve future decisions [74]. In repeated decisions with initially unknown reward distributions, a careful balance must be struck between learning about relatively unknown options (exploration) and gaining high immediate rewards (exploitation). It is crucial to consider both the estimated value of each option and the uncertainty associated with these estimations for resolving the exploration–exploitation dilemma optimally [54]. As Bayesian learning quantifies uncertainty, it provides a framework for studying how humans resolve this dilemma. Computational models and behavioral data in bandit tasks show that uncertainty influences human learning, attention, and exploration. Bayesian theories of cognition are supported, and subjective uncertainty is fundamental to understanding and decision making [74].

In uncertain circumstances, the expectations of results vary widely, and the outcome depends on those expectations. Subjective probabilistic inferences provide information for updating decision-making procedures under uncertainty [75]. A decision-making criterion with uncertain trade-offs induces inconsistent present preferences.

Subjective probabilistic inference involves different levels of information acquisition, which plays a role in everyday forecasting cases. Forecasting results constrain cognitive processes and influence decision-making procedures [76]. Participants with low probabilistic inference overestimate or underestimate future unknown rewards as uncertainty increases, resulting in an enhanced fear of losses, which becomes an obstacle to acquiring information. Risk preference is not critical in decision making under unknown uncertainty. Time inconsistency and cognitive ability are related to unknown uncertainty [75].

The “information” versus uncertainty puzzle is solved by the CDP, which views variability as a mechanism that helps functionality in dynamic environments. Per the CDP, variability, inherent to all systems, also characterizes behaviors and is a mechanism for improved functionality under uncertainty [77]. The CDP views human behavior and functionality similarly to other biological systems. It considers a degree of variability, which can change based on internal and external factors. However, the CDP does not consider consciousness and human aspirations, mainly due to insufficient knowledge about the biological mechanisms underlying them [77,78]. Thus, behaviors and the decision-making process inherently involve variability and constraints, which continuously change, leading to fluctuations in the decision process.

## 8. The CDP Defines Biological Variability, Adding to the Medical Challenges Posed by Biological Uncertainties

Per the CDP, the subject’s inherent biological variability and various environmental factors, as uncertainties, present significant challenges for diagnosis and treatment.

The limits of current medical technology and knowledge further exacerbate diagnostic, prognostic, and treatment uncertainties [79]. Predicting disease outcomes and treatment efficacy can be challenging in personalized medicine due to uncertainties. Numerous sources of uncertainty exist in medicine, ranging from the inherent variability of human biology to the uniqueness of each patient and the dynamic and unpredictable nature of diseases [80]. Genomics has revealed intricate details about genetic predispositions, but interpreting this information and its implications for patient care remains challenging. Climate change, habitat loss, and invasive species pose additional uncertainties. These environmental changes affect ecosystems and biodiversity in complex ways [48,81].

Diagnosis is the initial step of medical practice and is one of the most essential parts of complex clinical decision making. It is often accompanied by ambiguity and uncertainty. A single disease may show itself differently depending on the patient and with varying intensities. One symptom may be linked to different diseases. Patients with multiple diseases can interact and disrupt the typical presentation of any disease [82]. A patient presenting symptoms in medicine may simultaneously be attributed to various diseases and social processes.

During a clinical consultation, the goal is to diagnose a sign or complaint and determine an appropriate treatment to resolve the issue. However, it is common for physicians to approach clinical problems using a mental model based on Heisenberg’s uncertainty principle, whether they realize it or not. This approach is complicated by various factors, some of which may be clearly understood and others not [11]. It is challenging to account for this process’s variabilities and multifactorial uncertainties. Specific fixed points in the clinical process include gender, race, age, weight-to-height ratio, privilege, social life, and intelligence. However, some people age differently from others in their age group, and obesity may not always be a determining factor. Social factors are crucial at every stage. It is common for the focus to shift entirely to the diagnosis once made, but other elements continue to impact a person’s life and response [11,83,84]. Choosing the most appropriate treatment strategy is challenging without definitive personalized guidelines [66]. A therapeutic uncertainty arises due to unanticipated side effects or individual variations in response to treatment [84,85].

The CDP states that the inherent variability in all biological systems and the dynamic nature of these variabilities pose a significant challenge in accurately diagnosing and selecting appropriate therapy [86,87,88].

## 9. The CDP Refines Strategies for Dealing with Uncertainties in Biology

Per the CDP, variability is constrained and personalized, enabling proper function under continuously changing conditions; therefore, it must be accounted for by modelers (3). Biological noise and variabilities, inherent to all systems, must be recognized to improve strategies for dealing with uncertainties in these complex systems. The CDP provides a method for accounting for variability [4]. It provides methods for overcoming the challenges posed by inter- and intra-subject variability. CDP-based modeling considers the personalized dynamic variability of biological systems’ structures and functions by accounting for variability’s continuously changing nature. It is a mechanism for regulating functionality in dynamic internal and external environments. CDP-based digital twins are designed to improve the accuracy and predictability of these modeling systems by accounting for the inherent variability [28].

Data analytics and computational models allow for the analysis of large datasets and the simulation of biological processes. They enable complex biological interaction prediction and identification patterns using machine learning and AI63. Adaptive function in conservation biology can adjust strategies based on ongoing monitoring and learning. Flexibility in conservation plans allows for effective responses to uncertainties, enhancing conservation efforts [67]. Researchers can address uncertainties through cross-disciplinary collaborations by integrating insights from numerous fields. A personalized approach to healthcare is encouraged by recognizing and respecting each subject’s uniqueness. Including patients in decision making helps patients navigate uncertainty by integrating their values and preferences [89,90].

Studies indicate that these systems exhibit inherent variability in addition to technical variability. It is rare to replicate a time series of synthetic gut communities in chemostats, which leaves the question of whether stochasticity impacts gut community dynamics unanswered [91]. Using 16S rRNA for the community profiling of gut microbiomes resulted in high variability across replicate vessels and technical variability, whereas the variability was significantly lower for flow cytometric data [92]. These changes were accompanied by reproducible metabolic shifts, a rapid depletion of glucose and trehalose concentrations, and a decrease in formic and pyruvic acid concentrations within 12 h of switching to chemostat mode. According to 16S rRNA gene sequencing, the observed variability in the synthetic bacterial human gut community is mainly due to technical variability. Flow cytometry and HPLC data show low variability, suggesting a highly deterministic system [92].

This example highlights the need to distinguish biological variability from testing variation.

## 10. CDP Is Utilized to Refine Fuzzy Logic Methods to Address Biological Uncertainties

Fuzzy logic is a tool for describing and representing biological or medical scenarios in which states and outcomes are not entirely true or wholly false but somewhat accurate or partially false [93]. It introduces partial truth values between “true” and “false” [82]. It is a qualitative computational approach that provides a method to model and manage uncertainty and reduce ambiguity. It can predict diseases and generate patient warning statuses with reliable results [94,95]. Fuzzy logic offers an approach to address the two types of biological uncertainty [96,97]. It provides various modeling approaches, including linear, nonlinear, geometric, dynamic, and integer programming. These approaches determine the optimal point under ambiguous conditions, giving decision makers a more flexible option [98]. Studies have shown the geometrical interpretation of fuzzy sets as points in a fuzzy hypercube and provided two concrete illustrations in medicine and bioinformatics [82].

Fuzzy logic approaches, such as fuzzy Petri nets (FPNs), address various challenges in modeling uncertain biological systems [96]. A study assessed the specific type of biological system that can be described using ordinary differential equations or continuous Petri nets (CPNs). Combining CPNs and fuzzy logic created an algorithm for simulating fuzzy continuous Petri nets (FCPNs). It models biological systems where specific kinetic parameters are unavailable or their values vary due to environmental factors [99].

Whole-cell modeling aspires to depict a cell’s species and biochemical processes within a single model. It necessitates the integration of various modeling methods, including stochastic (for stochastic processes), deterministic (for continuous components), and fuzzy (for different sources of uncertainties) methods into a single model. Incorporating various types of FPNs to address a range of uncertainties in a biological system may entail adding fuzzy logic to existing stochastic/deterministic hybrid Petri nets. By employing this approach, results from hybrid models, which integrate structural and kinetic uncertainties, can be obtained in a given context [43,96].

Developing synthetic communities under well-controlled conditions is essential to understanding the mechanisms driving community dynamics [28,100]. Exposure assessment is based on predictive models specifying growth parameters for each microbe species [101]. The effect of biological variability on the outcome of exposure assessment was determined by accounting for microbial growth variability among strains of a single species. Each parameter of the exposure assessment, growth parameters, and shelf-life conditions was described by a probability distribution describing variability and uncertainty. Variations within species affected the exposure assessment result [102]. Uncertainty handling and visualization can be incorporated into the original data during data processing. Ensuring that all stages of the pipeline are aware of uncertainty and capable of propagating it is crucial. When visually representing uncertainty, it is vital to differentiate between explicit and implicit distributions [103]. Fuzzy logic has been integrated into medicine to address these uncertainties. Fuzzy clustering techniques were developed concerning the complex decision-making systems of human crowds and other biological organisms’ behavior [104,105].

In medicine, diagnosing diseases can be complex due to uncertainties and inaccuracies. While conventional statistical methods have been used, they do not capture the gradual fluctuations within medical measurements. Fuzzy control charts provide a valuable alternative to address the complexities of health science. They offer a comprehensive view of medical data, thereby contributing to quality control in healthcare and enabling informed decisions for enhanced patient care [106,107]. Fuzzy logic systems accurately model medical conditions by converting imprecise data into understandable formats, replicating human reasoning through linguistic variables to improve symptom interpretation, diagnosis, and treatment [108]. Fuzzy linear programming applies fuzzy logic to medical decision making [109]. Examples of using fuzzy logic include predicting the response to the treatment of citalopram in alcohol dependence, analyzing diabetic neuropathy, detecting early diabetic retinopathy, calculating volumes of brain tissue from magnetic resonance imaging (MRI), and analyzing functional MRI data. Fuzzy logic can also improve decision-making in radiation therapy, control hypertension during anesthesia, and detect cancers [82,110]. It is well-suited for developing knowledge-based systems for disease diagnosis, selecting medical treatments, and the real-time monitoring of patients’ data. Various studies have utilized fuzzy expert systems to diagnose lung diseases, differentiate syndromes, and classify diseases [109,111].

Multiple-criteria decision analysis (MCDA) is an essential aspect of operational research, evaluating alternatives based on conflicting criteria in fields such as economics, medicine, government, and engineering. Making the best choice considering these criteria is challenging, but the MCDA technique simplifies the process. It is applied in medicine and healthcare, where multiple criteria come into play for evaluating alternatives in various scenarios and assessing alternatives with multiple conflicting criteria [109,112]. In medicine, decision making involves trade-offs between different, often conflicting, objectives. A well-structured approach to evaluating options with multiple criteria enhances decision-making quality [113,114].

Bioinformatics makes use of fuzzy logic. Bioinformatics uses computers to analyze biological data. These data can include genetic information, experimental results, patient statistics, and scientific literature. Bioinformatics uses tools to analyze and model large sets of biological data, manage chronic diseases, study molecular computing and cloning, and develop training tools for bio-computing systems [115]. Processing large volumes of biological data, often imprecise and ambiguous, calls for robust integrated bioinformatics systems. Fuzzy technology improves the adaptability of protein motifs, comparing variations between polynucleotides, analyzing experimental expression data, and aligning sequences based on a fuzzy reinterpretation of a dynamic programming algorithm. Other applications include DNA sequencing using genetic fuzzy systems, clustering genes from microarray data, and predicting protein subcellular locations from their dipeptide composition. Gene expression data are also analyzed using these methods [116,117]. A geometrical interpretation of fuzzy sets involves using points in a hypercube to represent related mechanisms in stroke and drug consumption [82].

However, fuzzy logic has challenges such as developing fuzzy rules, diverse interpretations of outputs, extensive medical data and expertise requirements, lack of generalizable results, and the need to run the program for each patient. Therefore, applying fuzzy logic in medicine is difficult without preprogrammed software for different conditions and primary clinician training [109,118].

Per the CDP, models must consider biological variability and dynamic boundaries, necessitating a dynamic algorithm. It implies the need to fine-tune fuzzy algorithms by distinguishing inherent noise in the biological system from technical noise, which may be caused by the measuring device or changing external factors. The accuracy and clinical applicability of the modeling results depend on the models’ ability to accommodate inherent variability.

## 11. Using the CDP to Refine Fuzzy Control Charts

CDP-based modeling accounts for the variability and does not attempt to reduce it. In contrast, implementing quality initiatives such as statistical process monitoring (SPM) to achieve quality objectives involves assessing, monitoring, and reducing process variability in health monitoring. Control charts and process capability analysis (PCA) are components of SPM. These methods have proven effective in monitoring hospital performance indicators such as mortality rates, pre-operative and post-operative issues, and hospital-acquired infections. Control charts are tools for tracking processes and detecting changes in the average and variability of a specific quality attribute. They are employed in quality control studies to evaluate qualitative and quantitative characteristics [119]. Conventional control charts are essential tools in quality management for detecting process shifts. These charts, such as “X-bar and Range” and “X-bar and standard deviation”, monitor variability and assess process stability by tracking parameter shifts over time [120,121]. In traditional charts, the process is assumed to follow a normal distribution with known parameters, and parameter estimation is carried out reliably. These charts work well when the data are precisely and accurately known. However, determining the exact process may only sometimes be feasible. When quality characteristics are expressed using linguistic terms, the traditional control methods become inadequate for assessing the process [122].

Traditional control charts have two decision lines, namely the upper control limit (UCL) and the lower control limit (LCL). These limits indicate process stability when sample points fall within them. If the points exceed these limits, the process is considered unstable. The effectiveness of control charts is evaluated based on their ability to detect process shifts [123]. This concept aligns with the CDP, which sets a dynamic range for each system function. However, according to the CDP, this range allows the system to adapt to internal and external changes. Therefore, a lack of or too much variability is linked to malfunctions [3].

Control charts can deal with uncertainty using fuzzy set theory (FST). The FST models uncertainty in natural language and finds application in optimization, automatic control systems, information systems, imaging systems, and decision making [107,124]. Fuzzy control charts use two approaches, namely probabilistic and membership. They rely on expert judgments and fuzzy attributes. They can monitor process parameters and combine fuzzy logic with SPM rules to reduce false alarms and enhance shift detection [107,125]. FST can conduct fuzzy moving-average control charts and fuzzy weighted-moving-average control charts for individual measurements, along with their process capability indices (PCIs). These techniques rely on the flexibility and robustness of FST to handle linguistic expressions and uncertainties, improving process monitoring and decision-making [107,126]. The fuzzy control chart has advantages over the conventional control chart because it works without the constraints of normality assumptions. Schematically, it aligns with the CDP, as it considers system variability.

Fuzzy control charts were used to analyze the variability in health monitoring processes, such as patient’s hematocrit levels. Hematocrit data are known for their dynamic characteristics and potential fluctuations, making it an ideal candidate for fuzzy control charts. Exploring the application of customized fuzzy control charts for individual measurements focused on the variability in the hematocrit dataset [107]. The exponential smoothing method in R software (version 1.0) was used to forecast hematocrit variability. Calculating fuzzy triangular numbers for hematocrit data involves taking daily periodic measurements to capture the inherent uncertainty of hematocrit values. It derives fuzzy values encapsulating a range, including potential fluctuations in hematocrit levels. These fuzzy triangular numbers are characterized by three essential components, namely a lower boundary, a central or peak value, and an upper limit, enabling the effective communication of the membership degree of hematocrit values within imprecise intervals and providing an understanding of the variability in hematocrit data. The analysis of fuzzy moving-range control charts emphasizes specific individuals whose hematocrit measurements consistently deviate from the expected range. The application of fuzzy control charts to hematocrit data has revealed significant deviations in hematocrit values, indicating potential problems with measurement quality [107,127,128]. In this process, three types of fuzzy control charts were utilized, namely the moving-average control chart (FMACC), the moving-range control chart (FMRCC), and the weighted-moving-average control chart (FWMACC). These charts systematically evaluate the variability of medical data, particularly the estimated hematocrit values, using the α-cut approach. The α-cut midrange values are carefully determined and analyzed in the construction process. Additionally, fuzzy PCIs measure process capability, providing insights into data quality and its implications for decision making. These control charts have fuzzy rules, enabling precise analysis and the interpretation of minor shifts [107,129,130,131,132].

According to the CDP, it is essential to distinguish between variations related to the measurement quality and the inherent variability of the targeted biological measurement. A straightforward method to make this distinction is to analyze repeated measurements taken within a short timeframe, which can provide information about the measurement-related variability. The use of fuzzy logic in algorithms is beneficial when dealing with uncertainty. When variability is essential to the system and necessary for its proper function, it is crucial to focus on fuzzy methods to accommodate this inherent variability. Creating a fuzzy decision tree that considers this variability can reduce uncertainty and improve the accuracy of the outcomes. This approach may reveal new types of classes, reduce the number of unknowns, and make the algorithm output more biologically relevant.

## 12. CDP-Based Artificial Intelligence Platforms Assist in Navigating Unknowns and Overcoming Uncertainties, Which Constitute a Method for Using Variability and Regulating It

The CDP defines systems’ functionality and efficiency by their range of variability bounded by the dynamic borders. These borders determine the degree of variability, enabling adaptation to noisy environments. It is a built-in mechanism to deal with uncertainties and continuous perturbations. Per this principle, biological and environmental noises are regarded as uncertainty. By accepting them to a degree that optimizes function, systems can adequately adapt and accomplish their tasks more effectively [19].

The CDP-based artificial intelligence systems use biological variability to improve the functionality of biological systems. The platform comprises three levels. In the first level, variability is artificially introduced into interventions to improve diagnosis and treatment. It is an open-loop system, where noise is added to enhance the accuracy and efficiency of the outcome [28,66,86,88,100,133]. This system enables patients who lost the effectiveness of chronic medications by introducing variability into the treatment regimens within pre-defined ranges, both for the time of administration and the dosing, to regain the effectiveness of the therapeutic intervention.

The second level of the CDP-based platform is a closed-loop system, where the degree of variability in the intervention is modified based on the outcome. It enables the personalization of the algorithm. The system is dynamic, so the algorithm continuously implements personal changes. The platform’s third level quantifies variability signatures, such as genetic and immune parameters’ variabilities, and implements them into the algorithm output. It enables one to account for biological variability and other uncertainties in a personalized way [66,100]. This method enables the use of noise to improve the functionality of systems [38,77,78,91,133,134,135,136,137,138,139,140,141,142,143,144].

Per the CDP, system malfunctions indicate a dysfunction in the boundaries of variability, resulting in either an excess or a lack of the essential variability for proper functioning. It leads to an increase in uncertainty within the systems. The second-generation artificial intelligence platform based on CDP is designed to address these challenges by regulating variability dynamically and personally.

## 13. Conclusions

The CDP defines all systems based on their inherent variability and provides a platform for examining the drivers of variability, enabling the reintegration of biology. Biological variability uncovers fundamental principles that influence diversity across the biological levels of an organization [57]. Although variability differs from uncertainty, per the CDP, it is viewed as a form of built-in uncertainty, representing a regulated mechanism for efficient functionality. It fosters an interdisciplinary approach by providing a single law to all systems in the universe. Addressing uncertainty involves incorporating knowledge from different fields and leveraging technological advancements. In the medical field, professionals must adopt a culture of ongoing learning, research, patient-focused care, and ethical decision making to navigate uncertainties in diagnoses, treatments, and new diseases [145]. Future studies are designed to dissect some of the mechanisms that underlie the effects of CDP-based interventions for improving the functionality of systems by accounting for system variability.

## Data Availability

All data are available in public domains.

## Abbreviations

CDP	Constrained Disorder Principle
